# Carriers for the targeted delivery of aerosolized macromolecules for pulmonary pathologies

**DOI:** 10.1080/17425247.2018.1502267

**Published:** 2018-07-26

**Authors:** Nashwa Osman, Kan Kaneko, Valeria Carini, Imran Saleem

**Affiliations:** a School of Pharmacy and Biomolecular Sciences, Liverpool John Moores University, Liverpool, UK

**Keywords:** Aerosol devices, inhaled macromolecules, lung delivery, microcarrier, nanocarrier, nanomedicine, lipid-based systems, passive and active targeting, pulmonary pathologies

## Abstract

**Introduction:** Macromolecules with unique effects and potency are increasingly being considered for application in lung pathologies. Numerous delivery strategies for these macromolecules through the lung have been investigated to improve the targeting and overall efficacy.

**Areas covered**: Targeting approaches from delivery devices, formulation strategies and specific targets are discussed.

**Expert opinion**: Although macromolecules are a heterogeneous group of molecules, a number of strategies have been investigated at the macro, micro, and nanoscopic scale for the delivery of macromolecules to specific sites and cells of lung tissues. Targeted approaches are already in use at the macroscopic scale through inhalation devices and formulations, but targeting strategies at the micro and nanoscopic scale are still in the laboratory stage. The combination of controlling lung deposition and targeting after deposition, through a combination of targeting strategies could be the future direction for the treatment of lung pathologies through the pulmonary route.

## 1. Introduction

Pulmonary pathologies are main causes of morbidity and mortality worldwide, such as respiratory infections, chronic obstructive pulmonary disease (COPD), lung cancer, and asthma. Lung delivery of macromolecules through different carrier systems has gained increasing attention for the treatment of respiratory diseases, and this review aims to provide a concise, up-to-date overview of recent advances in delivery strategies of macromolecules via inhalation to treat pulmonary pathologies.

### 1.1. Pulmonary route for inhaled macromolecule drug delivery

**Figure 1.**
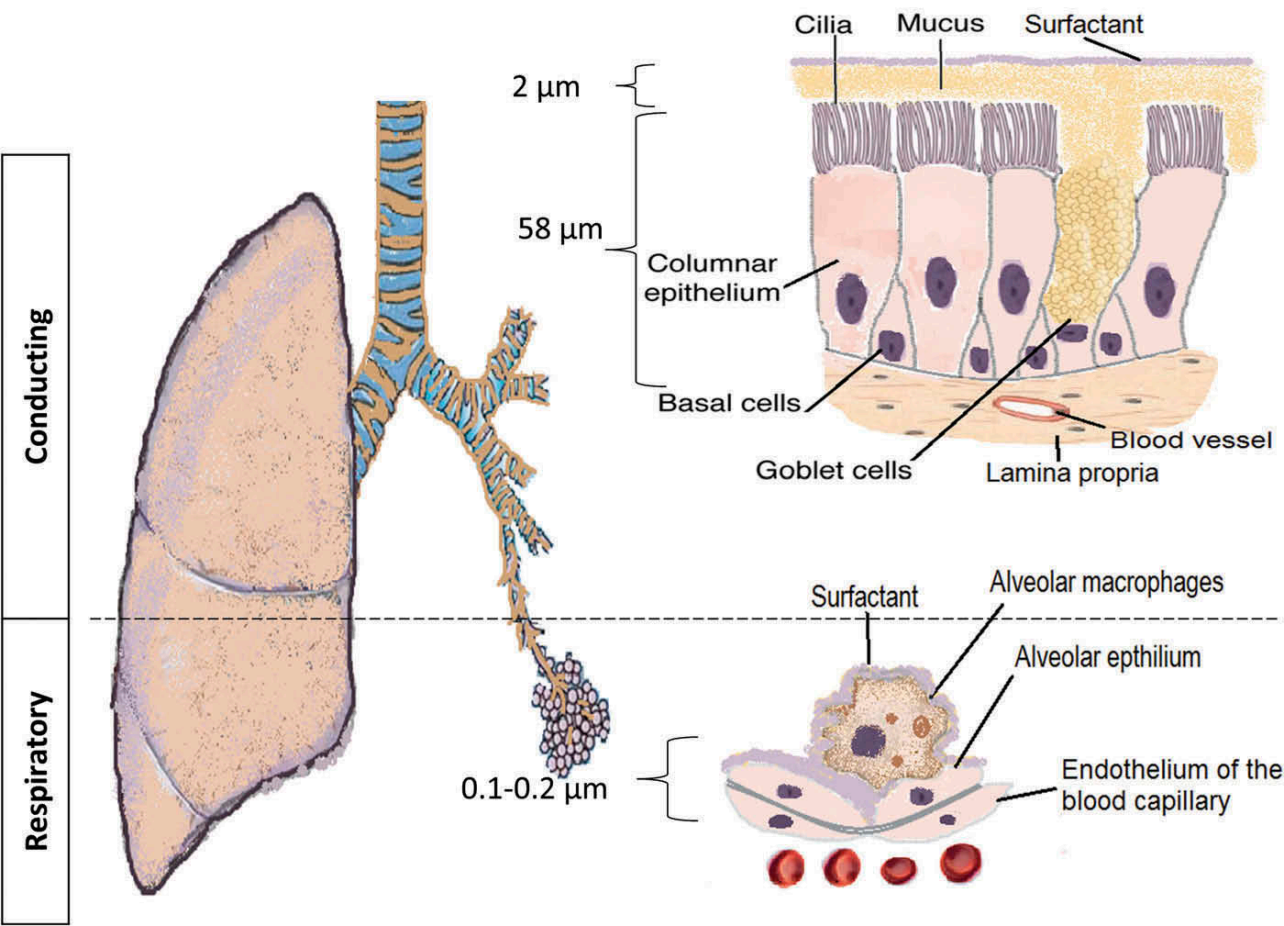
The pulmonary barrier structure in the conducting and respiratory airways.

The pulmonary route is composed of two structural parts that have different physiological characteristics (Figure 1): the conducing airways and the respiratory airways. The conducting or upper airways extend from the nose, pharynx, trachea to the terminal bronchioles a series of approximately 16 generations. This is followed, by the respiratory bronchioles starting at approximately 17 generations and ending at alveolar sacs. The conducting part has pseudo-stratified columnar ciliated epithelium with mucous secreting cells, which are responsible for the mucociliary clearance of the airstream from any exogenous particles, dust, and bacteria. The thickness of the epithelium is approximately 60 µm with a thick mucus layer lined with a lung surfactant layer [1]. The surface area is about 2 m^2^. The main functions are to transport the air to the gas-exchange area and to humidify, adjust the temperature, and filter of the incoming air stream.

There are numerous factors that can contribute to the sub-optimal delivery of aerosolized carriers or macromolecules in the conducting airways. One factor is the deposition of aerosolized matter against the walls of the airway. The size, shape, and density of particles can influence the deviation of particle flow from the streamlines and result in subsequent impaction away from the main absorptive alveolar surface. Another factor is the humid internal environment (approaches 90%), which can increase of the size of the hygroscopic particles and favors their deposition and early mucociliary clearance [2]. Underneath, the tight junctions between the epithelial cells presents as another barrier for carrier and macromolecule flux. The conducting airway is associated with common diseases such as asthma, chronic obstructive pulmonary disease (COPD), cystic fibrosis (CF), and emphysema. The pathophysiology is variable according to the disease conditions leading to bronchoconstriction and increase in the pulmonary tension with resistance to the air flow.

The respiratory airways are distal to the terminal bronchioles (branching around eight times) ending in alveolar sacs. There is a very thin (0.2–2 µm thickness) epithelium of alveolar cell type I (main cells) and type II secreting lung surfactant layer. The wide surface area (100–140 m^2^) is responsible for gas exchange and shows the highest permeability to water and macromolecules making it suitable target for drug delivery. Tight and gap junctions are the main connections between the alveolar cells [3]. A *blood–air* barrier formed from a single fused basal lamina of alveolar cells and endothelium, hosting a plethora of cells like dendritic cells (DCs), mast cells, and lymphocytes with secretory immunoglobulin. They not only play a role in antigen recognition and allergic reactions [4,5], but wandering alveolar macrophages that engulf foreign substances can be another barrier for carrier and macromolecule delivery. The absorption of deposited particles in the alveolar barrier is through either receptor-mediated transcytosis, paracellular passive transport via tight junctions, endocytosis, and engulfed by macrophages [6]. Size and molecular weight (MW) are important factors controlling the macrophage uptake; MW ≤ 25 kDa are rapidly cleared while MW ≥ 40 kDa are slowly cleared; whereas, phagocytosis is optimal for particle size of 1.5–3 µm. To escape the macrophage clearance mechanisms, drug particle and delivery systems must outside of the size range recognized by the macrophages [7]. For macromolecules, e.g. peptides, proteins, siRNA/miRNA, the lung surfactant may induce aggregation, and thus, potentially enhance macrophage clearance. Furthermore, the macrophages secrete peroxidases, inflammatory/immunomodulatory mediators and other host defense molecules that can degrade macromolecules and initiate a local immune response [8,9]. Disease conditions affecting the alveoli have serious percussions to the gas exchange, such as tuberculosis (TB), lung cancer, emphysema, pneumonia, acute respiratory distress syndrome (ARDS), and pulmonary edema. The pathophysiology of the respiratory tract and the severity of the disease are other limiting factors for successful macromolecule delivery.

### 1.2. Macromolecules and challenges for pulmonary delivery

The successful production and marketing of the first human recombinant insulin occurred over three decades ago as a protein drug, and subsequent development and production of macromolecule use for theranostic applications has been a rapidly evolving area of pharmaceutical industry [10]. Macromolecules are a heterogeneous group of proteins, including small peptides (20–30 amino acid residues, or called oligopeptides) (cytokines, enzymes, vaccines, monoclonal antibodies, and clotting factors), and genetic material (DNA, pDNA, RNA, siRNA, miRNA, ribozymes, and aptamers). They are molecules with superior drug-like properties, such as potency and specificity, due to highly selective receptor-binding that minimizes off-target side effects. However, due to their large size, hydrophilicity, MW, structural instability, and subsequent limited absorption at port of entry, their delivery has predominantly been conducted through injection. Short-circulatory half-life presents with another limitation, requiring frequent injections and consequently lowering the patient adherence to the treatment [11]. Aerosolized macromolecule delivery therefore has promising potentials as an alternative method for their delivery. Such potential benefits include non-invasiveness, possibility for self-administration, bypassing hepatic metabolism, and avoidance of the harsh proteolytic oral environment. Furthermore, the large surface area (100–140 m^2^ in humans) of the lungs with abundantly vascularized thin epithelium offers an encouraging route for local topical and systemic delivery. Increased bioavailability of the bioactive due to rapid onset of action, requiring smaller doses and reducing the potential of unwanted side effects, are amongst the advantages of macromolecule delivery via aerosol inhalation [12].

However, inhalation of macromolecules includes many challenges in their formulation, storage, and delivery [13]. Manufacturing and purification of macromolecules is a very costly process. Traditional methods of highly purified animal tissue protein extracts are hampered with immunogenicity and lack of specificity [14]. The newer recombinant macromolecule production lines are improving to increase the quantity and the quality of the macromolecules and reducing the cost, which is still very high. Using transfected mammalian cell lines, and recently, human cell lines, is more advantageous than *E. coli* clones with better similarity of macromolecules structure to the natural human proteins, albeit with high cost [14].

Macromolecules have unique structural features, and their activity is closely related to their structural integrity, which makes their formulation challenging. Different aerosol formulation approaches (e.g. dry powder, aqueous solution, liquid solution/suspension in a propellant vehicle) are incorporated to preserve their structural integrity. Most carrier-based formulations aim to encapsulate the macromolecules in their core to evade exposure to any enzymatic degradation. The carriers/formulations are equipped with different shielding to evade macrophages uptake, and targeting moieties can localize the active agent in the required site. Various stabilizers, absorption enhancers, and mucoadhesive adjuvants, such as fatty acids, surfactants, and protease inhibitors, have been utilized to promote higher bioavailability [9,15,16]. Furthermore, the optimal choice of aerosol delivery device is influenced by the macromolecule type, lung target site, and condition.

The safety profiles, including toxicity, local and systemic side effects, and immunogenicity, are important criteria in macromolecules evaluation, in addition to the efficacy and shelf life. In fact, many macromolecules have a major risk of inducing immunogenic reactions [17], with antibody production as the primary immune response. The therapeutic agent, after recognition as ‘foreign,’ is internalized, processed, and presented by antigen-presenting cells (APC), resulting in CD4 T-cell responses and the elevation in antibody titer. This immunogenic reaction can be used to our advantage and is sometimes targeted as in vaccine prophylaxis and therapeutics [5].

**Figure 2.**
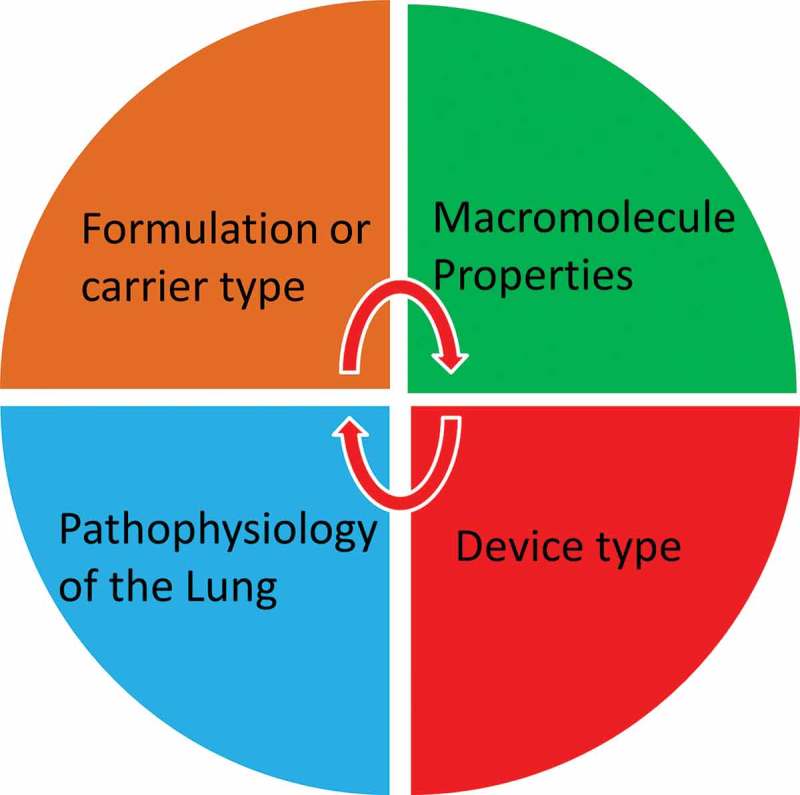
The interplay of factors for successful macromolecule delivery via inhalation.

The literature reports that many small RNA, such as siRNA, miRNA, and ribozymes, could be potential powerful therapeutics [18,19]. On the other hand, a wide variety of RNA species or analogues have also been reported to be immunogenic including ssRNA and dsRNA [20]. The investigation of the immune-compatibly represents one of the most important features for their translation to the clinic. A wide range of physicochemical properties such as MW, size, surface charge, solubility, and hydrophobicity play a role in macromolecule safety [17]. Hence, the interplay of macromolecule properties, formulation/carrier characteristics, device type, and the targeted lung disease are to be predetermined to achieve successful delivery (Figure 2).

## 2. Aerosolized delivery strategies for macromolecule drug delivery

Drug delivery to the lungs is achieved via respirable aerosols and can be used for topical as well as systemic delivery because of the advantages offered by the pulmonary route over other systemic routes of administration [9]. The aerosol is a stabilized dispersion of solid or liquid droplets suspended in a gaseous vehicle. The size of these droplets is always referred to as an aerodynamic diameter: the diameter of a sphere of unit density with the same settling velocity as the particle of interest. The inhalable portion of an aerosol is with an aerodynamic diameter below 10 µm and classified into coarse particles >2 µm, fine particle fraction (FPF) 0.1–2 µm, ultrafine fraction <0.1 µm [9]. Most of pharmaceutical aerosols are polydisperse but newer formulations and devices are developing monodisperse systems. It is generally accepted that a size range for pharmaceutical applications between 0.5 and 5 µm will allow deposition in the respirable airways [1]. The aerosol deposits the drug into the airways via different mechanisms mainly inertial impaction, gravitational settling, interception, and Brownian diffusion. The pharmaceutical aerosols that have a positive charge will be exposed to electrostatic precipitation (Figure 3A, B) [21,22]. A number of factors contribute to the deposition of aerosol particles within the airways; mainly particle characteristics such as size, shape, density, and surface charge, and the pathophysiology of the lungs. These factors are not only determinants for the quantity of the deposited particles but also to which regions of the respiratory airways are depositional targets [9]. It has been noted for optimal aerosol delivery, the conditions are aerosol particles with an aerodynamic diameter 0.5–5 µm and lung flow rates of 15–30 l/minute [21,23].

**Figure 3A.**
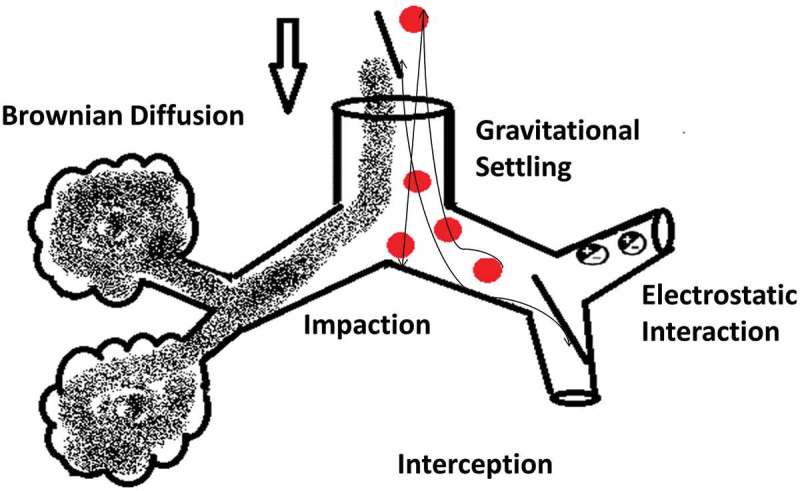
Particle properties and illustration of their deposition mechanisms.

**Figure 3B.**
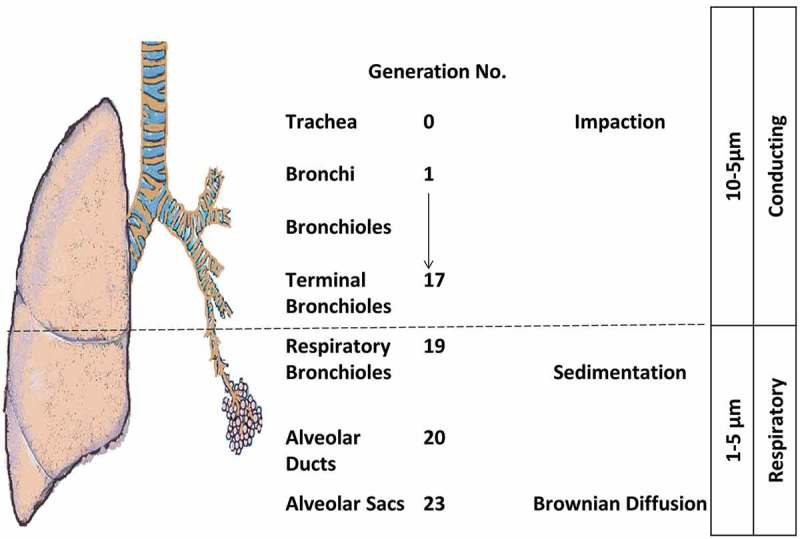
Particle properties and illustration of their deposition mechanisms.

### 2.1. Aerosol generating devices

Three widely known systems for aerosol drug delivery are nebulizers, metered dose inhalers (MDIs), and dry powder inhalers (DPIs). The choice of the device is multifactorial dependent on the active agent, the formulation characteristics, the target site, and the pulmonary pathophysiology. The main advantages and disadvantages of each device with recent insights for their development toward macromolecules delivery are represented in Table 1. Successful aerosol delivery to the lung should maintain high efficacy, dose-reproducibility, site-specificity, enhanced aerosol FPF, durability and stability for multiuse, simple handling, environmental-friendly, risk-free, and cost-effective treatment [1,24–29]. Examples of local aerosol macromolecules delivery to the lung are summarized in Table 2.

**Table 1.** Aerosol generating devices advantages, disadvantages, and recent developments.

Aerosol device	Advantages/disadvantages	Recent development for macromolecules delivery
**Nebulizers**: Deliver the drug or drug solution mixture.	**Advantages** No breath-hold Can be used by young, old or severely ill patients. Minimal breath training Drug concentration can be adjusted **Disadvantages** Expensive and bulky Nebulizers dose-delivery times are long Contamination risk Wet, cold mist Power source needed, electricity or compressed gas Less portable Main limitation for macromolecule delivery is unstability of proteins while the nebulization process.	Newer liquid-based aerosol generators are being developed to improve upon the conventional nebulization and increase the stability of active agents and the portability of the device. For example: Respimat® Soft Mist™ Inhaler (Boehringer, Germany): the device generates aerosol upon actuation without a propellant but pressurized by mechanical means. Surface Acoustic Wave (SAW) nebulizers: new devices that employ certain acoustic frequencies to generate liquid aerosols have proven efficient in macromolecule nebulization [30]. Omron Micro*Air*® VMT (Omron Healthcare, IL, USA): using the vibrating mesh aerosol-generating technology New systems are able to generate aerosols either under less harsh conditions; mechanically or through vibrating mesh. They are useful for treatment of asthma, CF, and COPD, for example, rhDNase nebulized enzyme for therapeutic treatment of CF
**MDIs**: Deliver the drug as liquid solution or suspension.	**Advantages** The most widely used oral inhalation device Mobility/or portability Short time required for delivery Deposition of up to 20% of released dose into the airways **Disadvantages** Hand-breath dependent Inability to adjust the drug concentration Failure of dose monitoring Difficulty to use may aggravate the patient incompliance. Poor solubility of macromolecule liquid droplets in a propellant vehicle (currently Hydrofluroalkane). Propellant allergy Poor aerosol characteristics and off target deposition in the upper airways. Expensive with new generations of MDIs	Developments of MDI to improve the patient ease of use, increase the FPF and lowering the off target deposition, and minimize the drug preparation without limiting their portability, and increasing their cost. The use of stabilizing or dispersing agents as sugars, surfactants, and ethanol during the formulation process improves the stability of macromolecules dispersed in the propellant and enhances the aerosol characteristics (FPF > 50%) and lowers oropharyngeal deposition Improvements in hand-breath coordination, portability Accessory devices that can improve the administration process: Bag: InspirEase, Holding chambers: Aerochamber, MediSpacer, ACE (Aerosol Cloud Chamber), Spacer: OptiHaler DNase has been successfully formulated and delivered through MDIs for CF [31].
**DPIs**: Deliver spray-dried or lyophilized drug as fine dry powders clouds.	**Advantages** Small portable devices No propellant No hand to mouth coordination Breath-actuated or energized aerosol generators do not require breath holding Short dose-delivery time No cold effect Countable doses and easily monitored ~ 12–40% of the emitted dose is deposited into the airways. Consistent stability with longer shelf life **Disadvantages** Only available for limited number of drugs, due to production costs associated with challenging formulation of the active agent as a dry powder that exhibits flow-ability, disperse-ability, and stability Breath-dependent might not have a sufficient inspiratory volume to stimulate the powder dispersion De-agglomerating challenges to produce inhalable aerosols Potential for off target deposition in the upper airways. Unit dose might require reloading prior each use Humidity might increase the size of hygroscopic particles favoring the deposition in upper airways and might affect the stability of macromolecules.	DPIs represent the popular choice for delivery of macromolecules through single dose devices [25]. Examples include HandiHaler™ (Boehringer Ingelheim, Germany), and Aerolizer™ (Novartis Pharma, CH). Multidose DPIs, i.e. Diskus™ (GlaxoSmithKline, UK) and Turbuhaler™ (AstraZeneca, Sweden), which are still being improved to enhance FPF of the generated aerosol. Inefficient breath-actuated DPIs have been improved into energized patient-independent devices, such as the Spiros™ (Dura Pharmaceuticals, CA, USA), that do not eliminate the flow-rate need that might affect the drug deposition.

**Table 2.** Examples of aerosolized carriers for macromolecule delivery for lung pathologies.

Indication or disease	Therapeutic macromolecule	Carrier/device	Type of study	Outcome/efficacy	References
Aerosolized vaccines	W-1 L19	PLGA NP	Preclinical *in vitro* cell assay	PLGA NPs were successfully formulated and loaded with W-1 L19 oligopeptides prepared from a highly immunogenic part VP2 capsid protein of Canine parvovirus (CPV). These NPs were successfully uptaken on J-774 cell lines with a non-toxic nitric oxide production and high immune response that could be a promising vaccine against CPV	[32].
	Ricin vaccine	Liposome	Preclinical *in vivo* mouse assay	A liposomal preparation of a natural toxoid A-chain of Ricin, which is natural Lectin from *Ricinus communis* plant that was successfully delivered by aerosols into mice lung.	[33]
	Pneumococcal surface protein A (PspA)	NPMP via DPI	Preclinical *in vivo* mouse assay	NPMPs particles as DPI *and in vivo* lung instillation in mice induced successful lung immunity.	[51]
	Tuberculosis (TB): Anti-TB vaccine Muramyl dipeptide	Instillation	Preclinical *in-vivo* mouse assay	*Mycobacterium bovis* Bacille Calmette-Guérin (BCG) was successfully intratracheal instillation in mice model of TB and compared to the subcutaneous route to find stronger mucosal lung immunity induced.	[34]
Acute respiratory distress syndrome (ARDS)	Surfactant Proteins	DPI, nebulization, instillation	*In vivo* animal study	Different surfactant proteins were delivered to the lung via aerosols as dry powders, nebulized or instillations in various *in vivo* animal models of acute lung injury.	[35–37]
Cystic fibrosis (CF) Primary ciliary dyskinesia (PCD)	A purified solution of recombinant human deoxyribonuclease (rhDNase), a mucolytic agent reducing mucous viscosity and secretions.	Nebulized or MDI	In clinics	Nebulized protein or MDIs [Approved by FDA, Genzyme NCT01712334]	[31]
Lung transplant	Cyclosporin A	Nebulized	Phase III clinical trails	Cyclosporin A was delivered via nebulized aerosols that showed high absorption and lung retention lung recipient in a randomized, double-blind, placebo-controlled clinical trial that showed improved the outcomes in chronic rejections.	[38]
Lung cancer and metastasis	Interleukin-2	Nebulized	Clinical trials phase III	Multicenter clinical trial showed high efficient delivery with low toxicity through nebulized aerosols.	[39]
	IgG1	Nebulized	*In vitro* study	Nebulized monoclonal antibodies against A431 cells showing promising results for lung cancer.	[40].
	Gene delivery: Akt1 siRNA	NP	*In vivo* animal study	Aerosol delivery of nanoparticle PEI of Akt1 siRNA significantly suppresses lung tumorigenesis in K-rasLA1 Mice.	[21]
Alpha-1-antitrypsin deficiency Emphysema CF	Alpha1 proteinase inhibitor (Alpha-1-Antitrypsin)	Nebulized	Clinical trials phase II/III	Nebulized Alpha-1-Antitrypsin was well tolerated in patient with CF in phase II/III clinical trials in Europe. (ClinicalTrials.gov: NCT01684410)	[41]

### 2.2. Carrier delivery systems

A carrier delivery system allows an active agent to be stably encapsulated or adsorbed, aiming to improve the pharmaco-kinetics and -dynamics. The carriers can be conveniently used to improve the stability, prevent macromolecule degradation, increase cellular uptake, and confer efficient targeting to the site of action with a homogenous distribution and favorable retention, with a reduced side effect by shielding (Figure 4A) [24]. The carriers are ideally biocompatible, biodegradable, and non-immunogenic, with high stability and scalability [21,42]. The carrier selection is dependent on interplay of factors mentioned in Figure 2. For successful aerosol delivery of macromolecules, preconditioned understanding of macromolecule and aerosol characteristics, target pathophysiological lung condition (inspiratory flow, lung volumes, breath-holding), and the suitability and stability of the macromolecule aerosol generated by nebulization, MDIs, and DPIs is required [1].

**Figure 4A.**
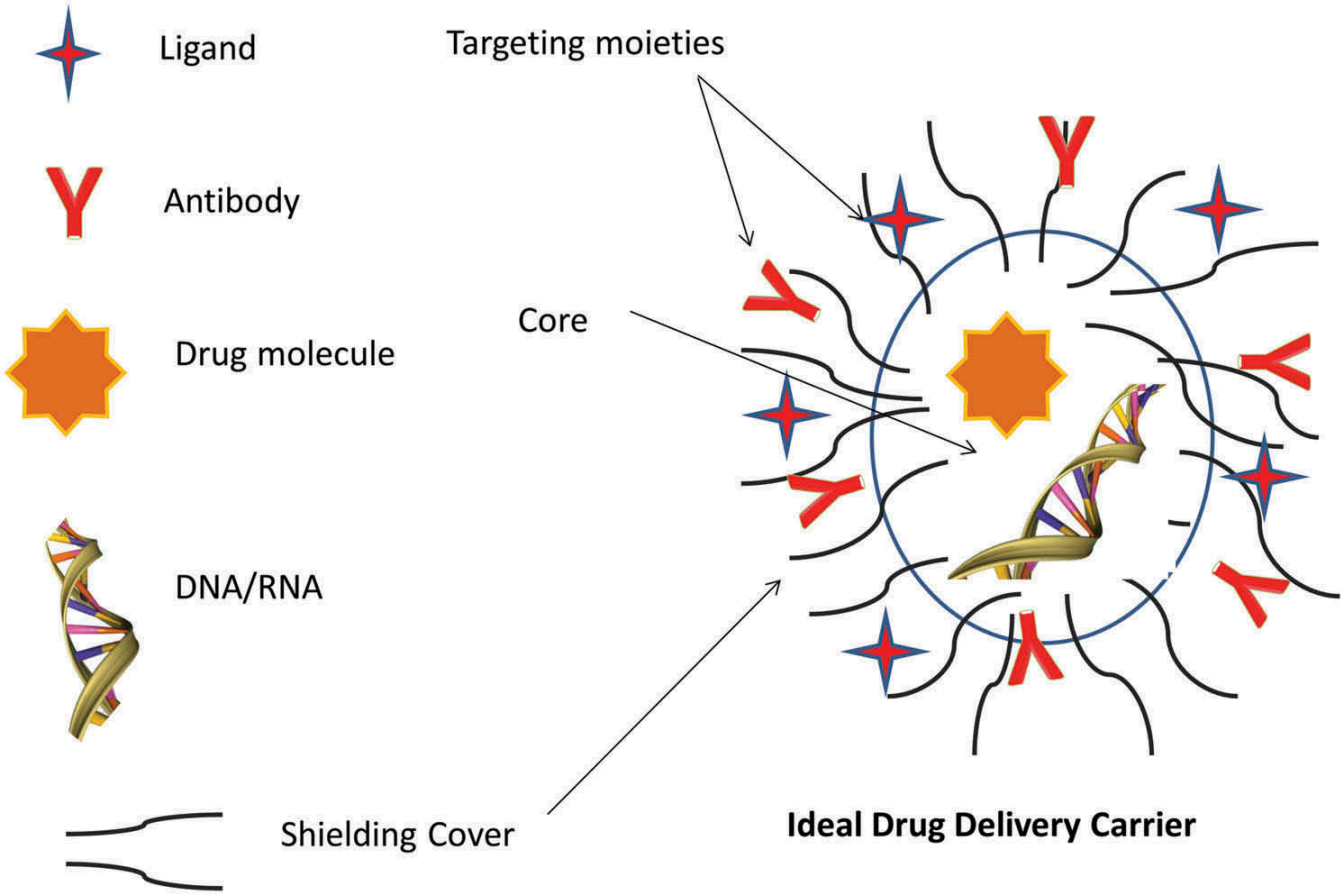
Ideal drug delivery carrier.

#### 2.2.1. Polymeric particulate carriers

##### 2.2.1.1. Microparticles and nanoparticles

Although the defined size ranges for microparticles (MPs) and nanoparticles (NPs) can differ based on convention and sources [43], NPs will be referred to particles within the nanometer scale (1–1000 nm) and MPs as particles in the micrometer scale (1–1000 µm) for the purpose of discussing pharmaceutical carrier formulations in this review, NPs and MPs have a variety of physicochemical properties, such as size, surface area, shape, molecular weight, porosity, hydrophobicity and charge, that allow modifications and functionalization to suit a wide range of macromolecules (Figure 4B) [24,44]. These carriers offer high loading capacity, protection from enzymes resulting in improved macromolecule stability with enhanced lung distribution and retention. Furthermore, they can be actively targeted to the site of action and have mechanisms for controlled release with a net gain of reduced dosing frequency and improved patient compliance. Moreover, they can be easily formulated from a wide variety of natural and polymeric biodegradable and biocompatible materials such as chitosan, alginate, poly Lactic-co-Glycolic acid (PLGA), polyethylenimine (PEI), and poly (l-lysine) (PLL) [45]. However, the formulation processes to produce NPs and MPs from the above polymers include harsh steps and solvents that can potentially denature the macromolecule or alter its structure such as emulsification/solvent evaporation, spray dryer, freeze dryer, and supercritical fluid technology [46,47].

**Figure 4B.**
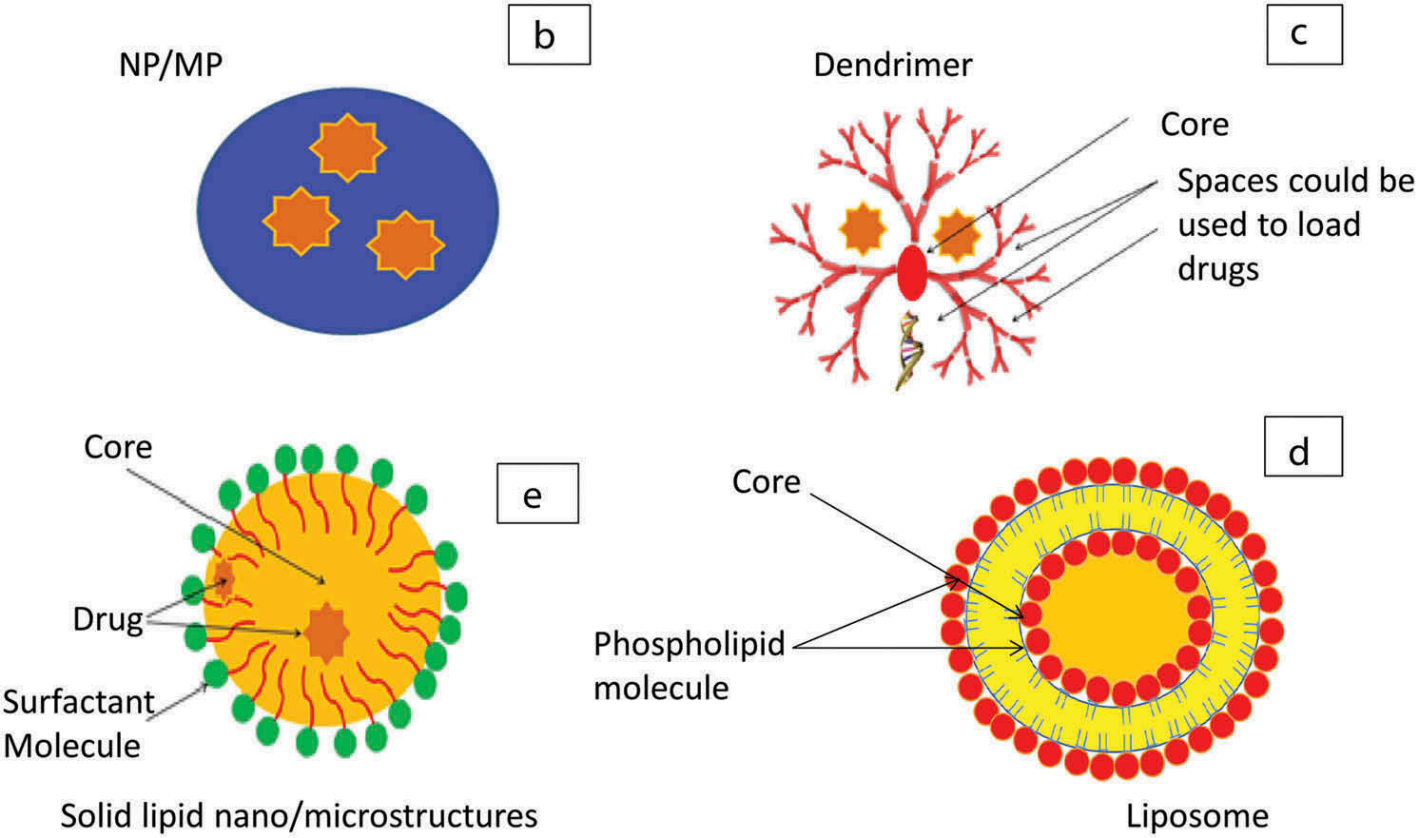
Different types of macromolecule carriers. B: NP/MP, C: dendrimers, D: liposomes, E: solid lipid structures.

Prior optimization steps are required to improve loading and stability. For example, surface coating or functionalization improves the pharmacokinetics; particle shielding with lipids or hydrophilic polymers as polyethylene glycol (PEG) lowers the macrophages uptake and recognition, mucoadhesive particles increase the lung retention, i.e. chitosan, coating of carriers with active targeting ligands such as antibodies will be discussed later [44]. A study showed a promising PEG-co-polyester MPs delivery system, composed of co-block polymer poly(glyceroladipate-co-ω-pentadecalactone) incorporating PEG within the polymer chain, as a suitable carrier for α-chymotrypsin, a mucolytic enzyme, demonstrating high loading efficiency, retention of enzyme activity and suitability for DPI  [48]. It is also difficult to deliver NPs in their single particulate form due to their high aggregation properties that favor their clearance. Consequently, NPs have been incorporated within microcarrier systems via spraydrying using sugars and amino-acids into nanoparticles embedded within microparticles (NPMPs) [49]. Their main advantages are evading the mucociliary and macrophages clearance. Upon their delivery to lower airways, the MPs degrade releasing the NPs that contain the active agent. They show successful DPI formulation with both good flow-ability and dispersibility. Kunda et al., showed successful polymeric cationic NPs formulation loaded with a Bovine Serum Albumin (BSA) as a protein model and was developed into DPI with good biocompatibility against A549 cell line [50]. Furthermore, they successfully developed a NPMPs loaded with pneumococcal surface protein A (PspA) as DPI vaccine against pneumococcal infection that successfully induced antibody response against *in vitro* DCs line [50]. Recently, this work was continued by Rodrigues et al., which demonstrated successful vaccination antibody production through *in vivo* study via lung instillation in mice compared to subcutaneous injection [51].

Cationic polymers are receiving increasing interest as non-viral vectors for gene transfer such as PLL, PEI, chitosan, and dextran, although the transfection rates are still low compared to viral/plasmid vectors [21,24,50,52]. However, they are less immunogenic with anti-degradation shielding. In addition, their cationic charge may play a role in the improved cellular uptake as well as increase the risk of local toxicity due to the interaction with cell membrane, so extensive optimization is prerequisite. A double-blinded clinical trial of PEG-PLL NP-mediated gene delivery for CF, where a gene was transfected though nasal mucosa, showed promising results [53].

##### 2.2.1.2. Dendrimers

Dendrimers are highly branched polymeric carriers that have terminal amine, carboxyl, or hydroxyl groups and their size is within nano-scale (Figure 4C). These groups provide a platform for functionality with different targeting and therapeutic molecules. Their branched structure effectively encloses active (hydrophilic or lipophilic) agents. They have improved physicochemical properties based on biocompatible and biodegradable polymers. Uniform size with low polydispersity, stability, versatile ability for modifications, and amine group-transfection ability enhances their carrier properties [44,54]. However, the complex formulations with multifaceted steps to access the core and build branches are limiting factors. Polyamidoamine (PAMAM) dendrimers of different sizes were tested *in vitro* (Calu-3, primary alveolar cell lines), *ex vivo* (instillation into perfused rat lungs) to show successful uptake intracellular with no aggregation with the lung fluid. They showed promising results for biocompatible targeting the lung via inhalation [55].

#### 2.2.2. Lipid-based carriers

Lipid-based carriers are efficaciously used in aerosolized macromolecules delivery offering high stability of the formulation and long storage life. They provide protection against enzymatic breakdown and mucociliary or phagocytic clearance with reduced toxicity and immunogenicity. They can provide controlled or sustained release with enhanced cellular uptake with intracellular cargo delivery and suitable for hydrophilic/lipophilic active agents, and can be functionalized with targeting moieties to certain cells [24,56].

##### 2.2.2.1. Liposomes

Liposomes are phospholipids assembled vesicles possessing an aqueous core with a size range within the nano to micrometer scale (Figure 4D). They can be formulated from natural or synthetic lipids mostly with neutral or anionic charge such as phosphatidylcholines, sphingomyelins, phosphatidylglycerols, and phosphatidylinositols [44]. However, cationic liposomes are produced using lipids with a positive charge and are used for gene delivery due to their capability for ionic interactions with DNA, siRNA/miRNA [24,44]. They form single bilayer or multilayers that can be used for the controlled release, encapsulate hydrophilic/lipophilic active agents, and improve loading capacity, versatile chemical functionalization, and active targeting. Liposomes are extensively investigated carriers for a wide variety of drugs and macromolecules and can be locally delivered via nebulization or DPI [24,57]. Lung deposition and retention can be targeted via modifications of the physicochemical characters such as size, charge, and formulation composition ratio and lipid type. Liposomes have been used as carriers for vaccines [58], nucleic acids such as siRNA, miRNA, lncRNA, DNA for respiratory conditions such as COPD, CF, and lung cancer. Although some of these applications are commonly administered via invasive routes, a number of cell and animal studies have proved their suitability for local lung delivery [56,59,60].

##### 2.2.2.2. *Solid lipid nano- and micro-structures (SLS*)

SLS are colloidal carriers having a core of solid lipids and a shell of biocompatible stable emulsifiers such as phospholipids, bile salts, and poly (vinyl alcohol) (Figure 4E) [61]. They offer improved stability, higher loading, and scalability, and shielding polymers are used to modify their surface reducing their phagocytic clearance. PulmoShperes™ are porous carriers that can be formulated from lipid-based emulsion spray dried coarsening with a shell of monolayers of phospholipids. They can incorporate the active agent in the core and deliver via inhalation device or instillation. They have been investigated in different clinical trials mainly for delivery of antibiotics but showing promising results for delivery of macromolecules as vaccine, peptides, and antibodies [62,63].

##### 2.2.2.3. *Archaeosomes (ARCs*)

ARCs are lipid-based carriers formulated from a polar type of lipids derived from archae membrane lipid of bacteria: *Sulfolobus acidocaldarius*. This lipid has di- or tetra-ether groups and has enhanced properties as gene carriers and macromolecules. They carriers are biocompatible and biodegradable with high stability cationic lipids with versatile functionality. ARC-DNA complexes were delivered via lung cell line A549 as model therapy for CF [64,65].

#### 2.2.3. Other carriers for pulmonary delivery

##### 2.2.3.1. Viral DNA/iRNA vectors

Viral vectors as gene transfection agent, such as retroviruses, adeno- and adeno-associated viruses, are well renowned. However, their use and investigation as carriers for pulmonary delivery is limited with majority of administration via parenteral route. Their main limitations are immunogenicity, limited loading, and poor scalability. Human CF transmembrane conductance regulator (CFTR) cDNA that was successfully loaded into adenovirus associate (tgAAVCF) had passed clinical trial phase I via nebulizers in CF patients, however was discontinued after failing to achieve the therapeutic targets in phase II [66]. Another study investigated the delivery of miRNA (Let-7) through lentivirus vectors in mouse lung cancer model resulted in tumor suppression against non-small cell lung cancer [67]. Plasmid–DNA complex have also been investigated using bacteriophages, such as bacteriophage ɸC31 integrase, to enhance the efficiency of delivery [66,68].

##### 2.2.3.2. Cell-based, biomimetic, and stem cells

Recent advances have enabled using cellular-based products for drug delivery purposes, such as using blood cells membrane. However, at present, no such aerosolized drug is found nonetheless that would be inspirational for future inhaled therapeutics [69].

## 3. General approaches for targeting pulmonary delivery of macromolecules

There are a number of approaches for targeting macromolecules and delivery vehicles to specific locations of lung tissue. Such targeted delivery in the lungs is an important aspect of increasing the concentration of drug to area of interest, which will ideally result in improved efficacy of drugs at the target site, as well as reduced systemic side effects. These methods for influencing the destination of active macromolecules can be categorized into passive and active forms of targeting, which refer to utilizing the unique physiological characteristics of the target environment, or specifically targeting molecules or components of the cell surface, respectively.

### 3.1. Passive targeting

The most common form of passive targeting within the lungs is by utilizing the discriminative tendencies of the airway physiology to influence the localization and deposition of the formulation. This bias and inequality in lung localization can arise initially due to how the delivery vehicle transits along the inhalation airflow and through the airways of the lungs. A widely utilized approach of passive targeting in the lungs is through control of particle size in the aerosol, which can be in the form of either suspended liquid or solid, to influence the aerodynamics; dictating the principal mechanisms of impaction. Such methods have been utilized in inhalation therapy for nearly 200 years and can be seen utilized in metered dose inhalers, nebulizers, and dry powder inhalers.

It is well established that aerosol particles with aerodynamic diameters in the range of 1–5 µm reach the lower parts of the respiratory tract; whereas, particles smaller than 1 µm do not deposit, and particles larger than 5 µm accumulate in the conducting airways [70]. Knowing that the deeper alveolar regions of the lungs exhibit greater drug absorption, as well as immune response in the case of immunotherapies, the optimum size range of 1–5 µm is generally the aim for contemporary pulmonary formulations. The particle physicochemical characteristics can be adjusted and utilized to achieve targeting to the desired lung region [71]. The targeted deposition in the lungs is an important mechanism for formulations such as particles containing itraconazole or amphotericin B for lung infections, and has been shown to increase the efficacy of the treatments [72]. Due to the concentration dependent effect of some antibiotics, it is important to have targeted delivery to ensure high concentration only at the affected sites to ensure optimum effects while avoiding side effects. Although small molecules such as antibiotics and airway therapeutics make up the majority of the delivered material, macromolecules such as siRNA have been heavily considered for pulmonary delivery [73].

Nanoparticulate formulations, such as polymeric NPs or liposomes, can exhibit controlled drug release at the site of delivery, leading to maintenance of therapeutic concentrations in the lungs for a longer duration [74]. Another unique property of NPs is the tendency to be phagocytized by alveolar macrophages, if they are in the range of 500–3000 nm [75,76]. The simultaneous effects, of NPs uptake and release, were demonstrated simultaneously, when a study of multilamellar liposomes containing rifampicin and isoniazid exhibited presence in alveolar macrophages and could maintain therapeutic concentrations in the lung tissue for 5 days [77]. Antimicrobial delivery via liposomes to the lungs has been shown to be beneficial in the case of cystic fibrosis and exhibits a controlled and sustained release, and some are currently in phase II clinical trials [78]. Studies involving the delivery of macromolecules such as siRNA have also been conducted using nanocarriers, as summarized more in depth elsewhere [73].

Other than aerosols, liquid micro volumes can also be administered by an airway catheter or bronchoscope to target specific areas of the lungs, but is generally thought to be invasive. Recent development of the delivery of micro-volumes of a liquid plug, by controlling liquid volume and air ventilation, has been hypothesized to be a potential approach for the targeted delivery of precise drug dosages to specific areas in the lung to treat pathologies such as cystic fibrosis and lung cancer [79].

Passive targeting in the lung can be summarized as changing the characteristics of the delivery vehicle to influence localization and uptake based on the physiology of the lung and cellular composition. Optimizing the characteristics of the aerosolized particles comprises a large aspect of this targeting approach as seen in the numerous aerosol delivery devices (Table 1).

### 3.2. Active targeting

Targeting specific tissue locations, cells, and molecules on cells and cell compartments, to increase the concentration of therapeutic macromolecules at the precisely desired location, is the hallmark aim of active targeting. One of the ubiquitous approaches for active targeting generally involves the conjugation of the carrier or macromolecule to a targeting ligand, which is generally in the form of protein and peptides, polysaccharides, glycolipids, glycoproteins, or antibodies. Cell surface receptors may be overexpressed on specific cell groups according to the pathologies such as cancer, or expressed on a particular cell group that are useful targets for therapy [80].

For lung cancer, several targets, which can be used to discriminate between healthy and cancerous cells, have been discovered. The epidermal growth factor receptor (EGFR) is known to be highly expressed in non-small cell lung cancer, and studies have been conducted investigating nano-formulations with EGFR targeting ligands for the delivery of cisplatin to the cancer tissue following inhalation [80]. These EGFR targeting particles were able to reduce tumor volume more effectively, as well as increase the bioavailability of cisplatin in the lung compared to the free drug [80]. Another target for lung cancer is carbonic anhydrase IX, which is expressed on tumor cells. Carbonic anhydrase IX antibody was incorporated onto liposome particles with CPP33, which is a cell-penetrating peptide. These liposomes loaded with Triptolide were shown to exhibit anti-tumor effects and reduced systemic side effects when administered endotracheally. Luteinizing hormone releasing hormone (LHRH) receptors are also known to be overexpressed on lung cancer cells and have been successfully investigated for selective targeting [81]. Although CD44 receptors have been suggested as another potential target, investigation using nanoparticles formulated with hyaluronan in an *in vivo* biodistribution study found that intrapulmonary nebulized administration did not result in increased accumulation of the nanoparticles in the tumors [82].

In addition to targeting tumor cells and pathogens, there are a number of immune cells that can be targeted for certain lung pathologies. In the case of tuberculosis, alveolar macrophages are recognized as a major part of the pathogenesis and thus a viable target for the delivery of antimicrobial agents. The *Mycobacterium tuberculosis* resides in the alveolar macrophages and forms the basis for the initiation of inflammation and the formation of granuloma. Macrophages are found in great numbers in the lungs and naturally possess the tendency to phagocytose particles under 5 µm, which can be capitalized for targeting. Formulating antibiotics in nanoparticles has been shown to increase the duration of therapeutic antibiotic concentration in the lungs, and studies involving rifampicin, isoniazid, and pyrazinamide indicated greater efficacy. Furthermore to phagocytic tendency, alveolar macrophages have also been identified with specific targets, including tetrapeptide tuftsin (Thr-Lys-Pro-Arg), O-steroyl amylopectin (O-SAP), and macrophage scavenger receptors [83], as well as mannose receptors that are also in abundance on DCs.

Activated T cells in the lung are another cell type that can be targeted in the case of asthma. These cells exhibit a Th2 phenotype, which is strongly associated with inducing airway inflammatory responses and chemo-attraction of inflammatory cells. Receptors for transferrin are known to be overexpressed in activated T cells, and, in combination with polycations such as low molecular weight polyethylenimine, the conjugated form was investigated for use in asthma. Transferrin-polyethylenimine (Tf-PEI) could be used to selectively deliver small interfering RNA (siRNA) to activated T cells in the lung. SiRNA can be used to silence specific mRNA expression through incorporation into the host cell, and is a promising approach for not only asthma, but also other lung diseases such as cystic fibrosis, chronic obstructive pulmonary disease, and cancer. Another cell group associated with asthma is the DCs, which are antigen presenting cells integral to the initiating immune responses. Although no specific study has been conducted targeting DCs by the pulmonary route, DCs exhibit tendencies for nanoparticle uptake and several ligands such as DEC-205 have been identified [84]. These targeting possibilities could be useful in applications such as vaccines.

To target the general lung epithelium for conditions such as asthma, lung structure cells have been investigated as potential targets, as β2-adrenergic receptors can be commonly found on bronchial smooth muscle cells. siRNA conjugated to salbutamol, which is a commonly used β2-AR agonist for asthma, has shown to exhibit greater gene silencing compared to the non-conjugated siRNA. Another way of targeting the epithelial cells of the lungs is through the human polymeric immunoglobulin receptor (hpIgR) [85]. This approach is postulated to be useful in cystic fibrosis: delivering anti-proteases to the epithelial surface to counter the elastase released from neutrophils, which can otherwise result in unnecessary inflammation and subdued clearance.

An interesting approach for increasing delivery of drugs into the epithelial tissue was tested by the administration of VEGF in an aerosolized form to increase permeability of microvessels in the lungs [86]. It has been hypothesized that the subsequent parenteral administration of magnetized nanoparticles containing the active molecule could potentially accumulate at the bronchial luminal epithelium through increased vascular endothelial permeability. Another example of magnetic targeting is the use of magnetic gradient fields to direct aerosol droplets containing superparamagnetic iron oxide nanoparticles to the desired regions of the lungs in mice. In addition to the delivery of particles to specific areas of the lung, the particles themselves can be used to release the active at specific areas based on physical external stimuli such as light, ultrasound, heat, and electric or magnetic fields. An example of targeted release by external stimulus is the use of ultrasound-sensitive microbubbles for local release of drugs [87]. Microbubbles created from lung surfactants showed a threefold increase in targeted deposition of the drug compared to common lipid-only microbubbles.

In addition to the delivery of macromolecules to specific tissue and cells, intracellular organelles such as the mitochondria have also been investigated as pharmacological targets. The mitochondria has been proposed as a target for cancer, as it is integral for cell functioning, and involved in cell proliferation and apoptosis [88]. To target the mitochondria, a study investigated the use of histidine to assist with escape from endolysosomes after through the ‘proton sponge’ effect, which is essentially disruption of the endosomal membrane through osmotic swelling caused by the influx of protons [88]. Another approach for endosome escape was demonstrated by attachment of the active macromolecule to low molecular weight polyethylenimine, which has cellular uptake and endosomal escape properties, leading to relatively high gene transfer efficacy [89].

There are indeed a vast number of identified targets for various cells involved in lung pathologies, but only a limited number have been investigated for efficacy after pulmonary delivery (Table 3). Despite the limited studies specifically for pulmonary delivery, numerous targets on cells of lung tissue and the local immune system have been identified for active targeting, and it may only be a matter of time before these molecular and physiological targets are investigated further for the delivery of therapeutic macromolecules.

**Table 3.** Summary of targets investigated specifically for formulations administered by the pulmonary route for lung pathologies.

Disease	Target	Effect	Reference
Lung cancer	Carbonic anhydrase IX (CPP33 dual-ligand modified triptolide-loaded liposomes)	Rats that were endotracheally administered triptolide-loaded liposomal formulations exhibited enhanced triptolide-loaded anti-cancer efficacy and reduced concentration of TPL in systemic circulation without apparent systemic toxicity.	[90]
Lung cancer	SP5-52 peptide (conjugated to Gem-loaded SFNPs)	Targeted formulations exhibited higher survival rate, less mortality, and no sign of metastasis in a lung tumor model.	[91]
Lung cancer	Hyaluronic acid (HA)	*In vivo* biodistribution studies with intrapulmonary nebulized administration of nanoparticle formulated with hyaluronic acid did not result in increased accumulation in the tumors.	[92]
Lung cancer	High MMP9 concentration	Mesoporous silica-based nanoparticles functionalized with bioresponsive caps exhibited proteasome inhibitor release at high MMP 9 concentrations in a lung cancer cell line transfected with MMP9 cDNA.	[93]
Lung cancer	Luteinizing hormone releasing hormone (LHRH) receptors	Tumor-targeted local delivery by inhalation of anticancer drugs and mixture of siRNAs was exhibited in a mouse orthotopic model of human lung cancer	[94]
Lung cancer	Integrin avb3	*In vitro* tumor cell uptake study using RGDfk-histidine-PLGA NPs, suggested that receptor mediated uptake could be taking place.	[95]
Cystic fibrosis	Human polymeric immunoglobulin receptor (hpIgR)	Antiprotease delivery through conjugation to an antibody correlated with areas of hpIgR expression in the respiratory epithelium in an *in vivo* study.	[85]
Asthma	Beta2-adrenergic receptor (β2-AR	SiRNA conjugated to salbutamol exhibited greater gene silencing compared to the non-conjugated siRNA in an *in vivo* study.	[96]
Asthma	Transferrin receptor (TfR)	Biodistribution study of siRNA, transferrin-polyethylenimine polyplexes in an *in vivo* model confirmed efficient and selective delivery of siRNA to activated T cells.	[89]

## 4. Conclusion

This review discusses the approaches for macromolecule delivery in the lungs, and strategies for targeted delivery based on different lung pathologies. The initial deposition of the formulation is mainly dictated by the aerosolization and inhalation; whereas, the formulation in the various carriers can further influence the delivery of the macromolecule to specific cells such as immune cells and cancer cells. The targeted delivery of these macromolecules could potentially lead to therapy that has higher efficacy and lower side effects.

## 5. Expert opinion

Targeting the lung via non-invasive aerosolization of therapeutic macromolecules requires a profound understanding of the macromolecule’s physicochemical characteristics, the formulation and carrier methods, the aimed inhaler device, and the pathophysiologic lung conditions.

Macromolecules are a heterogeneous group of high molecular weight molecules (proteins, peptide-based molecules, and genetic material) with superior drug-like properties. The production of macromolecules is still a low yield high cost industry using mammalian cells. This also transfers to their use in developing formulations suitable for pulmonary delivery. Hence, the main challenges in their inhalation formulation development are heterogeneity, loss of their stability, poor loading capacity, susceptible for enzymatic, mucociliary and phagocytic clearances, immunogenicity, and toxicity. Carrier-based delivery systems aim to overcome these challenges.

Various carriers systems offer different physicochemical characteristics that might suit one type of macromolecule but not all. The carrier selection process is an active pool of research cross-matching optimization trying to optimize different macromolecules with different carriers under different formulation processes. Inclusive and vigorous characterization for each carrier and macromolecule-carrier combination is important to infer their clinical effectiveness and biocompatibility. It is a very tedious process involving *in vitro* and *in vivo* animal, then human, experiments. Few of them will be clinically translated. The scalability and reproducibility of the formulation is another challenge that needs consideration prior designing carriers and is a major flaw of current methods involving solvent evaporation and ionic gelation. Methods such as microfluidics, which can overcome these issues regarding scaling up, are more frequently being investigated to improve the translation of preclinical formulations to the market [97]. Targeting moieties are important strategies to functionalize the formulation with homing facility that will prevent the non-specific delivery and increase the therapeutic outcomes. The availability of biodegradable and biocompatible materials is fueling the development of new carriers that can be used for pulmonary delivery. NPs, MPs, dendrimers, Lipid-based liposomes, and many other carriers have shown successful intracellular cargo delivery of proteins, peptides, and DNA/RNAi. Currently, carriers based on viral or bacterial plasmids are seriously limited by their unfavorable immunogenicity, high costs, and low scalability.

The delivery of the aerosolized formulation via the inhaler device still forms a significant aspect of pulmonary delivery, and the choice of device is based according to the patient condition, as well as the type of formulation. Although nebulizers, pMDI, and DPI have many successful, well-established, market products, improvement is still required to use these devices for optimal delivery of macromolecules. It is important to consider approaches that can overcome issues such as low deposition, increasing the FPF, improving stability and shelf-life, operate breath-independent and patient-friendly, and allow for multiuse without limiting their portability.

Lastly, the pathophysiology of lung conditions is variable from one disease to another. Pre-formulation evaluation for the characteristics of the target lung disease and subsequent manipulation of the macromolecule-carrier-device combination is required to achieve the desired delivery site, distribution, retention with reduced toxicity and immunogenicity, and better patient compliance. There is currently little known regarding the differences brought by lung diseases affecting the deposition and retention of active agents, which could be an avenue for future research. A need to produce pulmonary delivery systems that can address these limitations for each pulmonary pathology is a future tendency with achieving both site- and cell-specific delivery.

## Article highlights

- Macromolecules for treatment of lung pathologies are heterogeneous and can range from proteins such as antiproteases in cystic fibrosis to genetic material such as siRNA in asthma.
- Aerosolization is the main form of delivering materials to the lungs.
- Particle-based carriers have been investigated for delivery of macromolecules, as they can be made with specific physicochemical properties to suit a variety of macromolecules and also allow for controlled release and targeting.
- Targeting can be in the form of passive or active targeting, referring to utilizing the unique physiological characteristics of the target environment, or specifically targeting molecules or components of the cell surface, respectively.
- Only a few molecular targets have been investigated for active targeting through the pulmonary route but a considerable number have been identified and could be possible avenues for future investigation.

This box summarizes key points contained in the article.

## References

[1] Siekmeier R, Scheuch G. Systemic treatment by inhalation of macromolecules principles, problems, and examples. J Physiol Pharmacol. 2008;59(6):53–79. 19218633

[2] Stocks J, Hislop AA. Structure and function of the respiratory system; developmental aspects and their relevance to aerosol therapy In: BisgaardH, O'Callaghan C, Smaldone GC, editors. Drug Delivery to the lung. New York: Marcel Dekkar, Inc. 2002. p.47-104.

[3] Mason G, Peters A, Bagdades E, et al Evaluation of pulmonary alveolar epithelial integrity by the detection of restriction to diffusion of hydrophilic solutes of different molecular sizes. Clin Sci. 2001;100(3):231–236. 11222107

[4] Nicod LP. Lung defences: an overview. Eur Respir Rev. 2005;14(95):45–50.

[5] de Heer H, Hammad H, Kool M, et al Dendritic cell subsets and immune regulation in the lung. Semin Immunol. 2005;17(4):295–303. 15967679 10.1016/j.smim.2005.05.002

[6] Hastings R, Folkesson HG, Matthay MA Mechanisms of alveolar protein clearance in the intact lung. Am J Physiol. 2004;286(4):L679–89. 10.1152/ajplung.00205.2003 15003932

[7] C A F, Vanbever R Preclinical models for pulmonary drug delivery. Expert Opin Drug Deliv. 2009;6(11):1231–1245. 19852680 10.1517/17425240903241788

[8] Gordon S, Read R Macrophage defences against respiratory tract infections. Br Med Bull. 2006;61:45–61. 10.1093/bmb/61.1.45 11997298

[9] Kleinstreuer C, Zhang Z, Donohue JF Targeted drug-aerosol delivery in the human respiratory system. Annu Rev Biomed Eng. 2008;10:195–220. 18412536 10.1146/annurev.bioeng.10.061807.160544

[10] Gibbons A, Cryan SA A dry powder formulation of liposome-encapsulated recombinant secretory leukocyte protease inhibitor (rSLPI) for inhalation: preparation and characterisation. AAPS Pharm Sci Tech. 2010;11:1411–1421. 10.1208/s12249-010-9500-2 PMC2974130 20839079

[11] Wagner AM, Gran MP, Peppas NA Designing the new generation of intelligent biocompatible carriers for protein and peptide delivery. Acta Pharm Sin B. 2018;8(2):147–164. 10.1016/j.apsb.2018.01.013 PMC5925450 29719776

[12] Kunda N, Somavarapu S, Gordon SB, et al Nanocarriers targeting dendritic cells for pulmonary vaccine delivery. Pharm Res. 2013;30(2):325–341. 23054093 10.1007/s11095-012-0891-5

[13] Depreter F, Pilcer G, Amighi K Inhaled proteins: challenges and perspectives. Int J Pharm. 2013 15 447(1–2):251–280. 23499756 10.1016/j.ijpharm.2013.02.031

[14] Picanco-Castro V, de Freitas MC, Bomfim Ade S, et al Patents in therapeutic recombinant protein production using mammalian cells. Recent Pat Biotechnol. 2014;8(2):165–171. 25185983 10.2174/1872208309666140904120404

[15] Hussain A, Arnold JJ, Khan MA, et al Absorption enhancers in pulmonary protein delivery. J Cont Release. 2004;94(1):15–24. 10.1016/j.jconrel.2003.10.001 14684268

[16] Jorgensen L, Nielsen H Delivery technologies for biopharmaceuticals: peptides, proteins, nucleic acids and vaccines. United Kingdom: John Wiley & Sons Ltd; 2009.

[17] Guo S, Li H, Ma M, et al Size, shape, and sequence-dependent immunogenicity of RNA nanoparticles. Mol Ther. 2017;15(9):399–408. 10.1016/j.omtn.2017.10.010 PMC5701797 29246318

[18] Small MD RNAs in transcriptional gene silencing and genome defence. Nature. 2009 22 457(7228):413–420. 19158787 10.1038/nature07756 PMC3246369

[19] Bartel DP MicroRNAs: genomics, biogenesis, mechanism, and function. Cell. 2004;116(2):281–297. 14744438 10.1016/s0092-8674(04)00045-5

[20] Liu L, Botos I, Wang Y, et al Structural basis of toll-like receptor 3 signaling with double-stranded RNA. Science. 2008;320(5874):379–381. 18420935 10.1126/science.1155406 PMC2761030

[21] Ray A, Mandal A, Mitra AK Recent patents in pulmonary delivery of macromolecules. Rec Pat Drug Deliv Formul. 2015;9(3):225–236. 10.2174/1872211309666150729122231 26219931

[22] Gangurde H, Chordiya MA, Baste NS, et al Approaches and devices used in pulmonary drug delivery system: a review. Asian J Phar Res Health Care. 2014;4(1):11–27.

[23] Depreter F, Pilcer G, Amighi K Inhaled proteins: challenges and perspectives. Inter J Pharm. 2013;447(1):251. 10.1016/j.ijpharm.2013.02.031 23499756

[24] Cryan SA Carrier-based strategies for targeting protein and peptide drugs to the lungs. AAPS J. 2005 24;7(1):E20–41. 16146340 10.1208/aapsj070104 PMC2751494

[25] de Boer A, Hagedoorn P, Hoppentocht M, et al Dry powder inhalation: past, present and future. Expert Opin Drug Deliv. 2017;14(4):499–512. 27534768 10.1080/17425247.2016.1224846

[26] Muralidharan P, Hayes D Jr., Mansour HM Dry powder inhalers in COPD, lung inflammation and pulmonary infections. Expert Opin Drug Deliv. 2015;12(6):947–962. 25388926 10.1517/17425247.2015.977783

[27] Zhou Q, Tang P, Leung SS, et al Emerging inhalation aerosol devices and strategies: where are we headed? Adv Drug Deliv Rev. 2014;75:3–17. 24732364 10.1016/j.addr.2014.03.006

[28] Hoppentocht M, Hagedoorn P, Frijlink HW, et al Technological and practical challenges of dry powder inhalers and formulations. Adv Drug Deliv Rev. 2014;75:18–31. 24735675 10.1016/j.addr.2014.04.004

[29] Sebastian P GW Hertel, Friess Wolfgang Protein stability in pulmonary drug delivery via nebulization. Adv Drug Deliv Rev. 2015;93:79–94. 25312674 10.1016/j.addr.2014.10.003

[30] Rajapaksa A, Qi A, Yeo LY, et al Enabling practical surface acoustic wave nebulizer drug delivery via amplitude modulation. Lab on a Chip. 2014 7;14(11):1858–1865. 24740643 10.1039/c4lc00232f

[31] Pressler T Review of recombinant human deoxyribonuclease (rhDNase) in the management of patients with cystic fibrosis. Biol Targets Ther. 2008;2:611. 10.2147/btt.s3052 PMC2727891 19707442

[32] Derman S, Za M, Es A, et al Preparation, characterization and immunological evaluation: canine parvovirus synthetic peptide loaded PLGA nanoparticles. J Biomed Sci. 2015 20;22(1):89. 26482775 10.1186/s12929-015-0195-2 PMC4617543

[33] Smallshaw J, Richardson JA, Vitetta ES RiVax, a recombinant ricin subunit vaccine, protects mice against ricin delivered by gavage or aerosol. Vaccine. 2007;25(42):7459–7469. 17875350 10.1016/j.vaccine.2007.08.018 PMC2049008

[34] C, PerdomoZedler U, Kühl AA, et al Mucosal BCG vaccination induces protective lung-resident memory T cell populations against tuberculosis. mBio. 2016;7(6):e01686–16. 27879332 10.1128/mBio.01686-16 PMC5120139

[35] Ruppert C, Kuchenbuch T, Boensch M, et al Dry powder aerosolization of a recombinant surfactant protein-C–based surfactant for inhalative treatment of the acutely inflamed lung. Crit Care Med. 2010;38(7):1584–1591. 20400897 10.1097/CCM.0b013e3181dfcb3b

[36] Rey-Santano C, Mielgo V, Andres L, et al Acute and sustained effects of aerosolized vs. Bolus Surfactant Therapy Premature Lambs with Respiratory Distress Syndrome Pediatr Res. 2013;73:639. 23403804 10.1038/pr.2013.24

[37] Walther F, Hernández-Juviel JM. Waring AJ Aerosol delivery of synthetic lung surfactant. PeerJ. 2014;27(2):e403. 10.7717/peerj.403 PMC4045332 24918030

[38] Iacono A, Johnson BA, Grgurich WF, et al A randomized trial of inhaled cyclosporine in lung-transplant recipients. N Engl J Med. 2006;354(2):141–150. 16407509 10.1056/NEJMoa043204

[39] Merimsky O, Gez E, Weitzen R, et al pulmonary metastases of renal cell carcinoma by inhalation of interleukin-2. Ann Oncol. 2004;15(4):610–612. 15033668 10.1093/annonc/mdh137

[40] Maillet A, Congy-Jolivet N, Le Guellec S, et al Aerodynamical, immunological and pharmacological properties of the anticancer antibody cetuximab following nebulization. Pharm Res. 2008;25(6):1318–1326. 18030605 10.1007/s11095-007-9481-3

[41] Gaggar A, Chen J, Chmiel JF, et al Inhaled alpha1-proteinase inhibitor therapy in patients with cystic fibrosis. J Cyst Fibros. 2016;15(2):227–233. 26321218 10.1016/j.jcf.2015.07.009 PMC4993024

[42] Merkel O, Zheng M, Debus H, et al Pulmonary gene delivery using polymeric nonviral vectors. Bioconjug Chem. 2012 18;23(1):3–20. 21999216 10.1021/bc200296q

[43] Boholm M, Arvidsson R, Definition A Framework for the terms nanomaterial and nanoparticle. NanoEthics. 2016 01 10(1):25–40.

[44] Agrahari V, Agrahari V, Mitra AK Nanocarrier fabrication and macromolecule drug delivery: challenges and opportunities. Ther Deliv. 2016;7(4):257–278. 27010987 10.4155/tde-2015-0012 PMC5565796

[45] Haas J, Ravi Kumar MN, Borchard G, et al Preparation and characterization of chitosan and trimethyl-chitosan-modified poly-(epsilon-caprolactone) nanoparticles as DNA carriers. AAPS PharmSciTech. 2005 10;6(1):E22–30. 16353959 10.1208/pt060106 PMC2750407

[46] Alfagih I, Kunda N, Alanazi F, et al Pulmonary delivery of proteins using nanocomposite microcarriers. J Pharm Sci. 2015;104(12):4386–4398. 26505151 10.1002/jps.24681

[47] Al-Fagih I, K. Alanazi F, A. Hutcheon G, et al Recent advances using supercritical fluid techniques pulmonary administration macromolecules via dry powder formulations. Drug Deliv Lett. 2011;1(2):128–134.

[48] Tawfeek H, Evans AR, Iftikhar A, et al Dry powder inhalation of macromolecules using novel PEG-co-polyester microparticle carriers. Int J Pharm. 2013 30;441(1–2):611–619. 23124106 10.1016/j.ijpharm.2012.10.036

[49] Ungaro F, d’Angelo I, Miro A, et al Engineered PLGA nano- and micro-carriers for pulmonary delivery: challenges and promises. J Phar Phar. 2012;64(9):1217–1235. 10.1111/j.2042-7158.2012.01486.x 22881435

[50] Kunda N, Alfagih IM, Miyaji EN, et al Pulmonary dry powder vaccine of pneumococcal antigen loaded nanoparticles. Int J Pharm. 2015 30;495(2):903–912. 26387622 10.1016/j.ijpharm.2015.09.034

[51] Rodrigues TC, Oliveira MLS, Soares-Schanoski A, et al Mucosal immunization with PspA (Pneumococcal surface protein A)-adsorbed nanoparticles targeting the lungs for protection against pneumococcal infection. PloS One. 2018;13(1):e0191692. 29360883 10.1371/journal.pone.0191692 PMC5779684

[52] Davies L, McLachlan G, Sumner-Jones SG, et al Enhanced lung gene expression after aerosol delivery of concentrated pDNA/PEI complexes. Mol Ther. 2008;16(7):1283–1290. 18500249 10.1038/mt.2008.96

[53] Konstan M, Davis PB, Wagener JS, et al Compacted DNA nanoparticles administered to the nasal mucosa of cystic fibrosis subjects are safe and demonstrate partial to complete cystic fibrosis transmembrane regulator reconstitution. Hum Gene Ther. 2004;15(12):1255–1269. 15684701 10.1089/hum.2004.15.1255

[54] Kesharwani P, Jain K, Jain NK Dendrimer as nanocarrier for drug delivery. Prog Polym Sci. 2014;39(2):268–307.

[55] Morris C, Aljayyoussi G, Mansour O, et al Endocytic uptake, transport and macromolecular interactions of anionic PAMAM dendrimers within lung tissue. Pharm Res. 2017;34(12):2517–2531. 28616685 10.1007/s11095-017-2190-7 PMC5736778

[56] Willis L, Hayes D, Mansour HM Therapeutic liposomal dry powder inhalation aerosols for targeted lung delivery. Lung. 2012;190(3):251–262. 22274758 10.1007/s00408-011-9360-x

[57] Pinheiro M, Lúcio M, Lima JL, et al Liposomes as drug delivery systems for the treatment of TB. Nanomedicine. 2011;6(8):1413–1428. 22026379 10.2217/nnm.11.122

[58] Schwendener RA Liposomes as vaccine delivery systems: a review of the recent advances. Ther Adv Vaccines. 2014;2(6):159–182. 25364509 10.1177/2051013614541440 PMC4212474

[59] Ibraheem D, Elaissari A, Fessi H Administration strategies for proteins and peptides. Int J Pharm. 2014 30 477(1–2):578–589. 25445533 10.1016/j.ijpharm.2014.10.059

[60] Hyde S, Southern KW, Gileadi U, et al Repeat administration of DNA/liposomes to the nasal epithelium of patients with cystic fibrosis. Gene Ther. 2000;7(13):1156–1165. 10918483 10.1038/sj.gt.3301212

[61] Martins S, Sarmento B, Ferreira DC, et al Lipid-based colloidal carriers for peptide and protein delivery–liposomes versus lipid nanoparticles. Int J Nanomedicine. 2007;2(4):595–607. 18203427 PMC2676808

[62] Weers J, Tarara T The PulmoSphere™ platform for pulmonary drug delivery. Ther Deliv. 2014;5(3):277–295. 24592954 10.4155/tde.14.3

[63] Bunjes H Structural properties of solid lipid based colloidal drug delivery systems. Curr Opin Col Inte Sci. 2011;16(5):405–411.

[64] Réthoré G, Montier T, Le Gall T, et al Archaeosomes based on synthetic tetraether-like lipids as novel versatile gene delivery systems. Chem Commun (Camb). 2007;20:2054–2056. 10.1039/b618568a 17713076

[65] Kaur G, Garg T, Rath G, et al Archaeosomes: an excellent carrier for drug and cell delivery. Drug Deliv. 2016;23(7):2497–2512. 25777339 10.3109/10717544.2015.1019653

[66] Geiger J, Aneja MK, Rudolph C Vectors for pulmonary gene therapy. Int J Pharm. 2010;390(1):84–88. 19825403 10.1016/j.ijpharm.2009.10.010

[67] van Rooij E, Kauppinen S Development of microRNA therapeutics is coming of age. EMBO Mol Med. 2014;6(7):851–864. 24935956 10.15252/emmm.201100899 PMC4119351

[68] Rudolph C, Schillinger U, Ortiz A, et al Aerosolized nanogram quantities of plasmid DNA mediate highly efficient gene delivery to mouse airway epithelium. Mol Ther. 2005;12(3):493–501. 16099412 10.1016/j.ymthe.2005.03.002

[69] Wang J Editorial for biomimetic nanoparticles for drug delivery. Acta Pharm Sin B. 2018;8(1):2–3. 29872617 10.1016/j.apsb.2018.01.003 PMC5985695

[70] Kuzmov A, Minko T Nanotechnology approaches for inhalation treatment of lung diseases. J Cont Release. 2015;219:500–518. 10.1016/j.jconrel.2015.07.024 26297206

[71] Smola M, Vandamme T, Sokolowski A Nanocarriers as pulmonary drug delivery systems to treat and to diagnose respiratory and non respiratory diseases. Int J Nanomedicine. 2008;3(1):1–19. 18488412 PMC2526354

[72] Le J, Schiller DS Aerosolized delivery of antifungal agents. Curr Fungal Infect Rep. 2010;4:96–102. 20502511 10.1007/s12281-010-0011-0 PMC2868999

[73] Youngren-Ortiz SR, Gandhi NS, España-Serrano S, et al Aerosol delivery of siRNA to the Lungs. Part 2: nanocarrier-based delivery systems. HHS Public Access. 2017;34:44–69. 10.14356/kona.2017005 PMC5381822 28392618

[74] Watts AB, Williams RO. Nanoparticles for pulmonary delivery. In: Smyth HDC, Hickey AJ, editors. Controlled Pulmonary Drug Delivery. New York: Springer; 2011. p. 335–366.

[75] Mansour H, Rhee Y-S WX Nanomedicine in pulmonary delivery. Int J Nanomedicine. 2009;4:299–319. 20054434 10.2147/ijn.s4937 PMC2802043

[76] Chono S, Tanino T, Seki T, et al Influence of particle size on drug delivery to rat alveolar macrophages following pulmonary administration of ciprofloxacin incorporated into liposomes. J Drug Target. 2006;14(8):557–566. 17043040 10.1080/10611860600834375

[77] Deol P, Khuller GK, Joshi K Therapeutic efficacies of isoniazid and rifampin encapsulated in lung-specific stealth liposomes against Mycobacterium tuberculosis infection induced in mice. Antimicrob Agents Chemother. 1997;41(6):1211–1214. 9174172 10.1128/aac.41.6.1211 PMC163888

[78] Weers J, Metzheiser B, Taylor G, et al A gamma scintigraphy study to investigate lung deposition and clearance of inhaled amikacin-loaded liposomes in healthy male volunteers. J Aerosol Med Pulm Drug Deliv. 2009;22(2):131–138. 19422313 10.1089/jamp.2008.0693

[79] Kima J, O’Neilla JD, Dorrellob NV, et al Targeted delivery of liquid microvolumes into the lung. PNA. 2015;112(37):11530–11535. 10.1073/pnas.1512613112 PMC4577144 26324893

[80] Lee W-H, Loo C-Y, Traini D, et al Inhalation of nanoparticle-based drug for lung cancer treatment: advantages and challenges. Asian J Pharm. 2015;10(6):481–489.

[81] Taratula O, Kuzmov A, Shah M, et al Nanostructured lipid carriers as multifunctional nanomedicine platform for pulmonary co-delivery of anticancer drugs and siRNA. J Cont Release. 2013;171(3):349–357. 10.1016/j.jconrel.2013.04.018 PMC3766401 23648833

[82] Jeannot V, Mazzaferro S, Lavaud J, et al Targeting CD44 receptor-positive lung tumors using polysaccharide-based nanocarriers: influence of nanoparticle size and administration route. Nanomedicine. 2016;12(4):921–932. 26724540 10.1016/j.nano.2015.11.018

[83] Chaudhuri G. Scavenger Receptor-Mediated Delivery of antisense mini-exon phosphorothioate oligonucleotide to leishmania-infected macrophages. Biochem Pharmacol. 1997;53(3):385–391. 9065742 10.1016/s0006-2952(96)00763-0 PMC3088079

[84] Cohn L, Delamarre L Dendritic cell-targeted vaccines. Front Immunol. 2014;5:255. 24910635 10.3389/fimmu.2014.00255 PMC4039009

[85] Ferkol Cohn LA, Phillips TE, et al Targeted delivery of antiprotease to the epithelial surface of human tracheal xenografts. Am J Respir Crit Care Med. 2003;167:10. 10.1164/rccm.200209-1119OC 12615618

[86] Babincova M, Babinec P Aerosolized VEGF in combination with intravenous magnetically targeted delivery of DNA–nanoparticle complex may increase efficiency of cystic fibrosis gene therapy. Med Hypotheses. 2006;67(4):1002. 10.1016/j.mehy.2006.05.001 16797874

[87] Sirsi SR, Fung C, Garg S, et al Lung surfactant microbubbles increase lipophilic drug payload for ultrasound-targeted delivery. Theranostics. 2013;3(6):409–419. 23781287 10.7150/thno.5616 PMC3677411

[88] Günther M, Lipka J, Malek A, et al Polyethylenimines for RNAi-mediated gene targeting in vivo and siRNA delivery to the lung. Euro J Phar and Biopharm. 2011;77(3):348–449. 10.1016/j.ejpb.2010.11.007 21093588

[89] Xie Y, Kim N-Y, Nadithe V, et al Targeted delivery of siRNA to activated T cells via transferrin-polyethylenimine (Tf-PEI) as a potential therapy of asthma. J Cont Release. 2016;10(229):120–129. 10.1016/j.jconrel.2016.03.029 PMC4886848 27001893

[90] Lin C, Zhang X, Chen H, et al Dual-ligand modified liposomes provide effective local targeted delivery of lung-cancer drug by antibody and tumor lineage-homing cell-penetrating peptide. Drug Deliv. 2017;25(1):256–266. 10.1080/10717544.2018.1425777 PMC6058720 29334814

[91] Mottaghitalab F, Kiani M, Farokhi M, et al Targeted delivery system based on gemcitabine-loaded silk fibroin nanoparticles for lung cancer therapy. ACS Appl Mater Interfaces. 2017;9(37):31600–31611. 28836425 10.1021/acsami.7b10408

[92] Luo Y, Wang X, Du D, et al Hyaluronic acid-conjugated apoferritin nanocages for lung cancer targeted drug delivery. Biomater Sci. 2015;3(10):1315–1416. 10.1039/c5bm00067j 26301700

[93] van Rijt SH, Argyo C, Bölükbas DA, et al Mesoporous silica-based nanoparticles for targeted delivery of proteasome inhibitors to the lung. Eur Respir J. 2013;42:57.

[94] Kuzmov A, Minkoab T Nanotechnology approaches for inhalation treatment of lung diseases. J Cont Release. 2015;219:500–518. 10.1016/j.jconrel.2015.07.024 26297206

[95] Chen R, Xu L, Fan Q, et al Hierarchical pulmonary target nanoparticles via inhaled administration for anticancer drug delivery. Drug Deliv. 2017;24(1):1191–1203. 28844172 10.1080/10717544.2017.1365395 PMC8241141

[96] Luo Y, Zhai X, Ma C, et al An inhalable β₂-adrenoceptor ligand-directed guanidinylated chitosan carrier for targeted delivery of siRNA to lung. J Cont Release. 2012;162(1):28–36. 10.1016/j.jconrel.2012.06.005 22698944

[97] Abalde-Cela S, Taladriz-Blanco P, de Oliveira MG, et al Droplet microfluidics for the highly controlled synthesis of branched gold nanoparticles. Sci Rep. 2018 5;8(1):2440. 29402918 10.1038/s41598-018-20754-x PMC5799180

